# Genetic Loci of Plant Pathogenic *Dickeya solani* IPO 2222 Expressed in Contact with Weed-Host Bittersweet Nightshade (*Solanum dulcamara* L.) Plants

**DOI:** 10.3390/ijms25052794

**Published:** 2024-02-28

**Authors:** Robert Czajkowski, Dorota M. Krzyżanowska, Daryna Sokolova, Łukasz Rąbalski, Maciej Kosiński, Sylwia Jafra, Aleksandra Królicka

**Affiliations:** 1 Laboratory of Biologically Active Compounds, Intercollegiate Faculty of Biotechnology of UG and MUG, University of Gdansk, A. Abrahama 58, 80-307 Gdansk, Poland; dorota.krzyzanowska@ug.edu.pl (D.M.K.); daryna.sokolova@ug.edu.pl (D.S.); aleksandra.krolicka@ug.edu.pl (A.K.); 2 Department of Biophysics and Radiobiology, Institute of Cell Biology and Genetic Engineering, National Academy of Sciences of Ukraine, 148 Academika Zabolotnoho St., 03143 Kyiv, Ukraine; 3 Laboratory of Recombinant Vaccines, Intercollegiate Faculty of Biotechnology UG and MUG, University of Gdansk, A. Abrahama 58, 80-307 Gdansk, Poland; lukasz.rabalski@ug.edu.pl (Ł.R.); maciej.kosinski@phdstud.ug.edu.pl (M.K.); 4 Laboratory of Plant Microbiology, Intercollegiate Faculty of Biotechnology of UG and MUG, University of Gdansk, A. Abrahama 58, 80-307 Gdansk, Poland; sylwia.jafra@ug.edu.pl

**Keywords:** *Erwinia chrysanthemi*, colonization, alternative plant host, Tn5, random transposon mutagenesis, qRT-PCR

## Abstract

*Dickeya solani*, belonging to the Soft Rot *Pectobacteriaceae*, are aggressive necrotrophs, exhibiting both a wide geographic distribution and a wide host range that includes many angiosperm orders, both dicot and monocot plants, cultivated under all climatic conditions. Little is known about the infection strategies *D. solani* employs to infect hosts other than potato (*Solanum tuberosum* L.). Our earlier study identified *D. solani* Tn5 mutants induced exclusively by the presence of the weed host *S. dulcamara*. The current study assessed the identity and virulence contribution of the selected genes mutated by the Tn5 insertions and induced by the presence of *S. dulcamara*. These genes encode proteins with functions linked to polyketide antibiotics and polysaccharide synthesis, membrane transport, stress response, and sugar and amino acid metabolism. Eight of these genes, encoding UvrY (GacA), tRNA guanosine transglycosylase Tgt, LPS-related WbeA, capsular biosynthesis protein VpsM, DltB alanine export protein, glycosyltransferase, putative transcription regulator YheO/PAS domain-containing protein, and a hypothetical protein, were required for virulence on *S. dulcamara* plants. The implications of *D. solani* interaction with a weed host, *S. dulcamara*, are discussed.

## 1. Introduction

Plants are an important habitat and a valuable source of nutrients and water for microbes [1,2]. To gain access to these resources, pathogenic bacteria have developed strategies to invade, colonize, and initiate successful infections within the plants [3,4,5]. Whereas biotrophic pathogens establish complex interactions with their living hosts to obtain nutrients from the viable cells, necrotrophs use brute force to kill the host and acquire compounds from the dead or dying tissues on which they feed [6,7,8].

In general, all bacterial necrotrophs follow a similar mode of pathogenesis, secreting virulence factors (effectors) to provoke the decomposition of plant cells and tissues, thereby facilitating host colonization and nutrient acquisition [9,10]. Broad-host necrotrophic bacteria can cause diseases in diverse crops, ornamentals, and non-crop plants. It is generally accepted that these pathogens do not need to specifically recognize the plant species and/or the kind of plant tissue before they invade and establish an infection [8,11]. In contrast, the virulence factors (effectors) produced by these bacteria include a vast range of compounds, enzymes, and toxins that inhibit the structural and functional features of many plant species in different families worldwide [12,13]. Furthermore, disease symptoms can often be triggered merely by the presence of effectors, even in the absence of viable pathogens. For example, experiments done under laboratory conditions demonstrated that plant tissues, when exposed to culture supernatants containing plant cell wall-degrading enzymes, readily exhibit disease symptoms [14].

Among necrotrophic bacteria, Soft Rot *Pectobacteriaceae* (SRP, bacteria belonging to *Pectobacterium* and *Dickeya* species, formerly known as pectinolytic *Erwinia* spp.) are known for their extremally broad host range as well as for their aggressiveness toward various plant species [15,16]. SRP bacteria can infect at least 35% of angiosperm plant orders, both dicot and monocot plants, including agriculturally relevant species cultivated in all climatic zones [17]. Potato (*Solanum tuberosum* L.) is the most economically important crop affected by SRP [18]; however, these bacteria can also be associated with a variety of different plants (e.g., secondary hosts, nonhost plants, ornamentals) and are found in various environmental niches outside plants, including surface water, aerosols, soil, and insects [19]. Several aspects of SRP-plant interactions merit more attention. For example, it remains unknown why the weeds infected with *Pectobacterium* spp. and/or *Dickeya* spp., while clearly hosts for SRP, often develop only minor symptoms, whereas the same SRP strains cause severe disease symptoms in nearby potato plants [20,21].

In our prior investigation, employing a random Tn5-based GUS reporter transposon mutagenesis approach, we identified a variety of *D. solani* Tn5 mutants that exhibited up-regulation of gene expression exclusively upon contact with the weed host [22]. The present investigation aimed to characterize bacterial genes in these Tn5 mutants coding for factors important for *in planta* fitness and virulence that are expressed exclusively during infection of a secondary host, a weed plant. Our study investigated the interaction of *D. solani* strain IPO 2222 [23], the type strain of the species, and an emerging SRP pathogen that is causing increasing losses in agriculture in Europe and elsewhere, with bittersweet nightshade (*Solanum dulcamara* L.), a weed plant closely related to potato (*Solanum tuberosum* L.) that is frequently found in potato fields and is known to harbor SRP populations under natural conditions [21,24,25]. With this approach, we hypothesize that, despite being acknowledged as a broad host necrotrophic pathogen, *D. solani* can specifically recognize the plant host during the early stages of infection and modify gene expression to maximize their relative fitness on these various host plants.

## 2. Results

### 2.1. Identification of the Tn5 Insertion Sites in D. solani Mutants

From 52 *D. solani* Tn5 mutants obtained in our former study that exhibited gene expression up-regulation exclusively in contact with *S. dulcamara* [22], 45 mutants were successfully recovered from the −80 °C bacterial stock (in-house bacterial collection). They were further analyzed in this study to better identify the genes involved and the virulence contributions of these loci. The genomes of the 45 mutants were sequenced to identify the transposon insertion sites (Supplementary Table S1). A single insertion of Tn5 per genome was observed for each of the 45 mutants. Interestingly, Tn5 targeted the same locus in the case of mutants M328 and M880 (A4U42_RS17270—encoding a glucarate transporter), M598 and M980 (A4U42_RS08155—encoding a malate permease), and mutants M620 and M940 (A4U42_RS06665—encoding DNA-protecting protein DprA), therefore limiting the number of genes from 45 to 42 individual genes screened. Assessment of the KEGG biochemical pathways corresponding to the 42 different transcriptional units induced in the presence of *S. dulcamara* enabled their assignment of some to the cellular pathways involved in primary bacterial metabolism, infection process, synthesis of secondary metabolites, polysaccharide and capsule biosynthesis, transcriptional regulation, and transport across the outer membrane. Some of the genes found on the screen encode hypothetical proteins with unknown functions. Of the 42 different *D. solani* IPO 2222 transcriptional units interrogated for their transcriptional organization, 16 were predicted to be transcribed as individual genes. In contrast, the other 26 were predicted to be components of various operons (Supplementary Table S1). Interestingly, three disrupted genes defined by mutants M69, M271, and M943 and two disrupted genes defined by mutants M115 and M255 were each predicted to be components of two independent operons.

**Figure 1:**
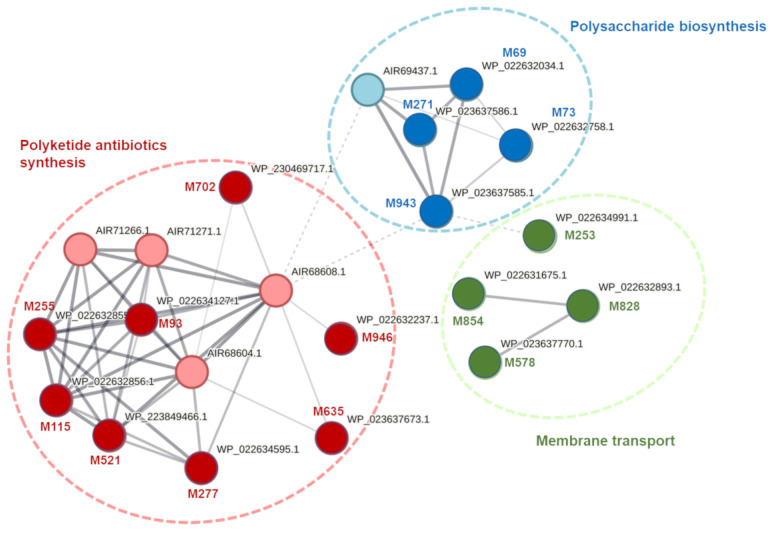
Protein networking for the 16 proteins of *D. solani* strain IPO 2222, whose expression was induced exclusively by the presence of *S. dulcamara* tissues. The analysis was performed using STRING with the proteome of *D. solani* strain IPO 2222 as a reference, and the image was manually curated. Each node represents a protein of IPO 2222 with a protein accession number. Nodes with dark color variants represent proteins whose expression was up-regulated, as evidenced by the GUS reporter assay [22], nodes with light color variants belong to the enriched group identified by STRING. Network-based on a full STRING network (the edges indicate both functional and physical protein associations). Network edges indicate confidence, line thickness indicates the strength of data support, and dotted lines represent clustering according to k-means. Interaction based on all available sources for STRING version 12.0, minimal interaction score = 0.4 (medium). Disconnected nodes were disabled. The M value near the node refers to the particular *D. solani* Tn mutant.

To gain more insights into the proteins encoded by the sequenced *D. solani* genes, these proteins were analyzed in silico for possible protein–protein interactions and protein networking. Protein network analysis, done with STRING, allowed the assignment of 16 analyzed proteins to three interconnected networks involved in (i) the synthesis of polyketide antibiotics, (ii) polysaccharide biosynthesis, and (iii) membrane transport systems (Figure 1). The other 26 proteins, although not assigned to networks, could be grouped into eight categories, including transport (Ds119, Ds273, Ds328, Ds564, Ds598, Ds652, and Ds1009), transcription and translation (Ds109, Ds241, Ds281, Ds468, and Ds890), sugar and amino acid metabolism (Ds321, Ds519, Ds573, Ds622, and Ds937), stress response (Ds327, Ds596, Ds620), envelope homeostasis (Ds278, Ds884), element homeostasis (Ds945, Ds955), phage-related (Ds585) and unknown proteins (Ds264).

### 2.2. The Capacity of D. solani Tn5 Mutants to Induce Symptoms in S. dulcamara Plants Cultivated in Culture Tubes

**Figure 2:**
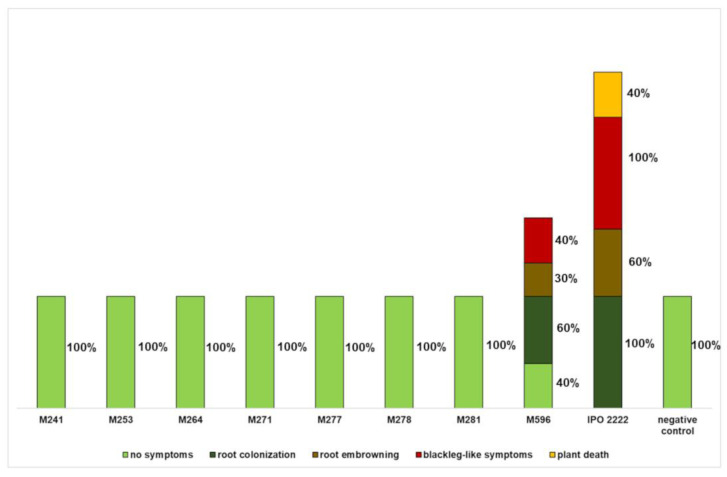
Disease symptoms observed after inoculation of the stem bases of *Solanum dulcamara* plants with eight *Dickeya solani* Tn5 mutants (M241, M253, M264, M271, M277, M278, M281 and M596), shown as the percentage of affected plants. Symptoms were evaluated at 14 days post inoculation. Percentages do not sum up to 100 as several symptoms per plant were observed. As a control, *D. solani* strain IPO 2222 (WT) was used.

All 45 Tn5 mutants were tested for their ability to cause symptoms in *S. dulcamara* plants grown in culture tubes in two independent experiments. No visible symptoms were observed in plants inoculated with sterile 1/4 Ringer’s buffer (negative control). In contrast, all plants inoculated with the WT strain expressed severe disease symptoms, leading to the death of the inoculated plants. Most *D. solani* Tn5 mutants tested in both experiments (77%) were virulent and caused disease symptoms comparable to those caused by the wild-type strain. However, seven Tn5 mutants (M241, M253, M264, M271, M277, M278, and M281) (raw genome sequences available in Supplementary Materials) did not cause any symptoms in *S. dulcamara* plants in either experiment, while mutant M596 (raw genome sequence available in Supplementary Materials) caused only reduced symptoms compared to the WT strain under the same conditions (Figure 2).

**Figure 3:**
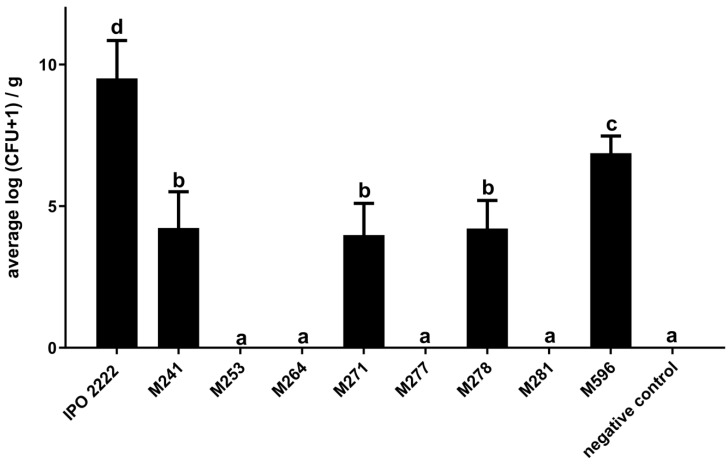
Population size of *D. solani* IPO 2222 (WT) and eight *D. solani* Tn5 mutants within stems of *S. dulcamara* plants after inoculation into stem-base. Results were considered significant at *p* = 0.05, and the pair-wise differences were obtained using the *t*-test. The means that do not share the same letters above each bar differ. Vertical lines represent standard deviation (SD).

The number of bacteria in haulms after stem base inoculation with mutants that expressed no symptoms (mutants M241, M253, M264, M271, M277, M278, M281) or reduced symptoms (mutant M596) was also evaluated. Haulms of plants inoculated with the WT strain harbored, on average, 10^9^–10^10^ cfu g^−1^ plant tissue. In contrast, no pectinolytic, neomycin-resistant bacteria were found in haulms of *S. dulcamara* plants inoculated with avirulent mutants M253, M264, M277, and M281 and only very low population sizes (10^4^–10^5^ cfu g^−1^ of plant tissue) of mutants M241, M271, and M278 were recovered. Population sizes of mutants that had exhibited reduced virulence were, on average, ca. 10^6^–10^7^ cfu g^−1^ of plant tissue (Figure 3).

### 2.3. Phenotypes of D. solani Tn5 Mutants

The eight Tn5 mutants that exhibited significant reductions or lack of symptom expression in *S. dulcamara* were checked in plate assays for phenotypes distinct from those of the WT strain. No differences were found between the mutants and the WT strain in all of the phenotypes tested with plate assays, including biofilm formation in vitro, generation time in rich and minimal media, motility, resistance to NaCl, growth under different temperatures, production of extracellular enzymes and resistance to H_2_O_2_. Likewise, most of the mutants did not differ from the wild-type strain in their ability to catabolize or tolerate various compounds when tested using BIOLOG GENIII and EcoPlate phenotypic microarrays. The collection of eight avirulent mutants differed from the wild-type strain in only 12 features of 94 tested with GENIII plates and 5 features of 31 tested with EcoPlate microarrays (Supplementary Table S2). Several mutants lost the ability to utilize various compounds as carbon sources or lost resistance to inhibitory compounds. Briefly, seven Tn5 mutants (all except M241) lost the ability to catabolize D-serine, three mutants (M241, M253, and M271) lost the ability to use L-glutamic acid, and two mutants (M253 and M271) lost the ability to use D-glucuronic acid for growth. Two other mutants (M253 and M264) lost the ability to use D-cellobiose as a sole carbon source. Mutants M271 and M278 lost the ability to survive in the presence of lithium chloride, and mutants M253 and M596 lost the ability to survive in the presence of guanidine hydrochloride. Individual mutants expressed several other phenotypes: mutant M278 lost the ability to grow at pH 5.0, and mutant M281 lost the ability to metabolize D-xylose and L-asparagine. Mutant M278 lost the ability to utilize D-mannitol, and M271 could not utilize L-serine. Simultaneously, some mutants gained phenotypes absent in the wild-type strain. Five mutants (M264, M271, M277, M278, and M596) gained the ability to use D-malic acid as a sole carbon source; three mutants (M241, M277, and M596) were able to grow in the presence of sodium butyrate; and three other mutants (M241, M277 and M596) gained resistance to fusidic acid. Two mutants (M277 and M596) could grow in the presence of 4% NaCl, and mutant M596 could grow in the medium containing 8% NaCl, unlike the IPO 2222 WT strain. Mutant M264 was able to utilize D-turanose for growth, a feature absent in the wild-type strain (Supplementary Table S2).

### 2.4. Expression of the Selected D. solani Genes in the Presence of S. dulcamara Analyzed with qRT-PCR

**Figure 4:**
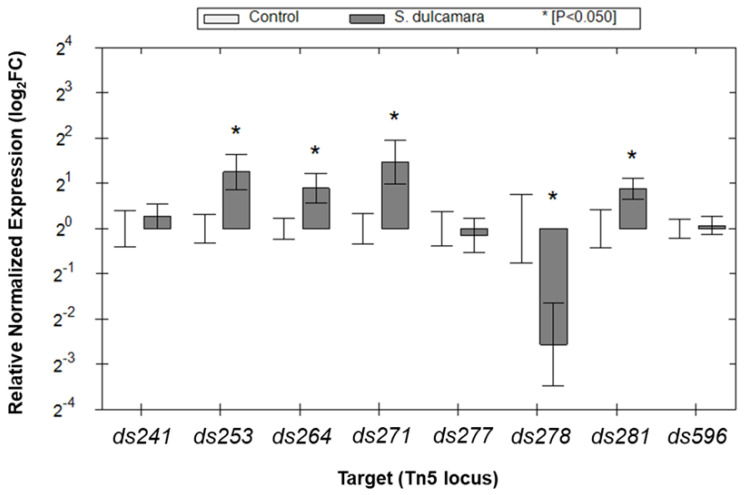
qRT-PCR analysis of the eight *D. solani* IPO 2222 loci found to be differentially regulated by *S. dulcamara* in a former transposon-based study. For each gene, we compared the expression levels in cells grown in a culture medium alone (control) to those in cells grown in a medium supplemented with in vitro-derived *S. dulcamara* plantlets. Genes *lpxC* and *yhb* were used as reference genes for data normalization. Per locus, the fold change under inductive to non-inductive conditions normalized to the expression of the control genes is shown [22]. Per locus, the fold change (log_2_FC) in gene expression of the target genes in the presence of *S. dulcamara* was calculated in relation to the control. Five biological replicates were analyzed per locus, and the results were averaged [22].

The eight candidate genes (*ds241*, *ds253*, *ds264*, *ds271*, *ds277*, *ds278*, *ds281*, and *ds596*) identified in mutants exhibiting the reduced ability to colonize plants and cause disease symptoms in *S. dulcamara* were subjected to gene expression analysis with qRT-PCR to better understand the qualitative assessments made earlier using *gus* reporter transposons [22]. Gene expression was compared between bacterial cells grown in a broth medium alone and in the same medium to which *S. dulcamara* seedlings, harvested from in vitro cultures, were added. Of the eight candidate genes, four genes (*ds253*, *ds264*, *ds271*, and *ds281*) showed statistically significant higher expression levels in the presence of *S. dulcamara* than under noninducible (control) conditions (Figure 4). In contrast, expression of gene *ds278* was decreased in the presence of *S. dulcamara* plant tissues (Figure 4). Overall, the relative transcriptional unit up/down-regulation of candidate genes, measured by qRT-PCR following 16 h of growth in the presence of *S. dulcamara*, ranged from approx. 3 times upregulation to 6 times downregulation (1.5 log_2_FC to −3 log_2_FC), depending on the particular transcriptional unit examined (Figure 4). The genes with increased expression in the presence of *S. dulcamara* encode proteins involved in LPS synthesis (*ds253*—*wbeA*), biosynthesis of the capsule (*ds271*—A4U42_RS11740, putative *vpsM* gene), and regulation of transcription (*ds281*—A4U42_RS17100, putative YheO transcriptional regulator/PAS domain-containing protein). The function of one of the induced genes, *ds264* (A4U42_RS11855), remains unknown. The down-regulated gene, *ds278* (A4U42_RS02215), encodes glycosyltransferase, but the process in which this particular glycosyltransferase is involved also remains unknown. Among the eight genes related to the virulence of *D. solani* on *S. dulcamara*, two viz. gene *ds271* (A4U42_RS11740) and gene *ds277* (A4U42_RS04070), encode capsular biosynthesis protein (WP_023637586) and DltB protein (WP_022634595), respectively, have homologs exclusively in other members of the Soft Rot *Pectobacteriaceae*, whereas six other genes have homologs ubiquitously present in different members of the *Pseudomonadota* phylum.

## 3. Discussion

The knowledge of strategies that necrotrophic SRP bacteria use to colonize and infect plant hosts other than potato remains scarce [14,26]. In our earlier study, we identified *D. solani* Tn5 mutants induced exclusively in the presence of a weed plant host [22]. This research aimed to examine bacterial genes mutated in these Tn5 mutants and responsible for factors crucial to *in planta* fitness and virulence, particularly those expressed solely during the infection of a secondary host—a weed plant.

As expected, most *D. solani* genes, whose expression was regulated by the presence of *S. dulcamara*, were involved in its interaction with this plant and/or with other environmental constituents. Of the 42 genes, 16 encoded proteins that could be cleanly grouped into three interconnected networks engaged in synthesizing polyketide antibiotics, polysaccharide synthesis, and membrane transport. The other 26 genes encoded proteins associated with, among others, transport, stress response, and sugar metabolism. This is perhaps not a surprise, as successful plant pathogens are expected to be able to cause infection in the plant under a variety of environmental conditions [3,27]. Likewise, the obtained results support earlier observations showing that for effective host infection, SRP bacteria need to adjust their metabolism to (i) successfully colonize the host and compete with other microbes for that niche, (ii) protect the cells from plant immune responses and other microorganisms present nearby, and (iii) produce virulence factors to facilitate host infection [28]. It is well established, therefore, that environmental modulation of gene expression is critical for SRP pathogenesis [29].

Many *D. solani* genes analyzed in our study did not individually have measurable effects on its virulence to *S. dulcamara* and probably represent genes involved in fundamental bacterial metabolism. It is noteworthy, however, that eight transcriptional units substantially influenced the virulence of *D. solani* in *S. dulcamara*. Surprisingly, of these eight genes, seven were not previously reported as encoding virulence factors used by SRP [28], revealing that many virulence traits in SRP bacteria and *D. solani*, particularly those required for infection by secondary plant hosts, remain to be elucidated.

The only *D. solani* gene found in this study that has been previously directly associated with the virulence of SRP was the protein UvrY (=GacA), which was disrupted in mutant M596. UvrY is a response regulator involved in a two-component signal transduction system found in certain bacteria, almost exclusively in the *Enterobacteriaceae* and *Pectobacteriaceae* families. UvrY regulates physiological traits influencing virulence, phage resistance, carbon metabolism, biofilm formation, stress resistance, quorum sensing, and secretion [30]. UvrY also impacts the expression of genes related to biofilm formation and motility—processes important for bacterial adaptation and survival. In *P. carotovorum*, UvrY homolog, protein ExpA (GacA) is responsible for transcriptional activation of the genes encoding plant cell wall-degrading extracellular enzymes, central effectors of the soft rot pathogens [31]. Similarly, in *D. dadantii* strain 3937, GacA is required for the appropriate production of virulence factors *in planta*, and a *gacA* mutant is impaired in causing disease symptoms [32]. A similar avirulence phenotype was found in our studies—*D. solani* mutant M596 expressed reduced virulence in *S. dulcamara* plants in vitro, indicating that GacA is also required for the virulence of *D. solani* not only on potato plants but also on *S. dulcamara*.

In contrast, among the seven other genes influencing *D. solani* virulence on *S. dulcamara* were those encoding tRNA guanosine transglycosylase Tgt (in M241), protein WbeA (in M253), hypothetical protein (in M264), capsular biosynthesis protein VpsM (in M271), DltB alanine export protein (in M273), glycosyltransferase (in M278), and putative transcription regulator YheO/PAS domain-containing protein (in M281). None of these genes have been previously characterized as virulence factors or primary fitness factors of SRP bacteria [28,33].

The gene encoding Tgt, a tRNA guanosine transglycosylase, was disrupted in mutant M241. Tgt is an enzyme critical in modifying certain tRNAs in various organisms, including bacteria [34]. It is primarily involved in the biosynthesis of queuosine, a modified nucleoside that is found in the anticodon loop of some tRNA molecules [35]. Queuosine modification is important for efficient and accurate mRNA translation, as it helps tRNAs recognize specific codons during translation [36]. It is speculated that queuosine modification plays a role in controlling biofilm formation and virulence in both Gram-positive and Gram-negative bacteria [37]. While alterations of tRNAs are known to affect the translation of virulence factors, including toxins and other secreted proteins, as well as the adhesion of bacterial cells to various surfaces [38], the direct connection between Tgt and the virulence of plant pathogenic bacteria has not been previously established [36]. It cannot be excluded that tRNA modifications could indirectly impact the virulence of SRP bacteria, including *D. solani*, by affecting the ability of the bacterium to produce specific proteins necessary for its direct interactions with plant cells or evasion of the host immune system [39].

The gene encoding WbeA was found to be disrupted in mutant M253. WbeA is associated with lipopolysaccharide (LPS) biosynthesis in Gram-negative bacteria, including *Dickeya* spp. and *Pectobacterium* spp. [40]. Lipopolysaccharides are crucial components of the outer membrane of these bacteria and play a role in the communication of bacterial cells with the environment [41]. Likewise, LPS is a recognized virulence factor of SRP bacteria [42]. LPS-defective mutants, as evidenced in our former studies [43], express lowered virulence and decreased fitness *in planta* [43], a phenotype confirmed in the current study.

The gene encoding VpsM, a capsular biosynthesis protein, disrupted in mutant M271, is an outer membrane protein having a beta-barrel structure involved in transporting the EPS precursors across bacterial outer membranes [44]. The protein is involved in the biosynthesis of the exopolysaccharide (EPS) capsule in bacteria, including some plant pathogens [45]. Although the specific role of the capsular biosynthesis protein in the ecology and virulence of *D. solani* is unknown, we speculate that its function in this bacterium is similar to the ones reported in other pathogens, including survival in the host environment and evasion of the host immune system [46].

The gene encoding DltB, alanine export protein was disrupted in mutant M273. DltB is a part of the Dlt (D-alanylation) system found in various bacterial species, including human pathogens [47]. Knowledge of the D-alanylation system has come mostly from studies on Gram-positive bacteria, including *Staphylococcus* and *Streptococcus* species, where it adds D-alanine to teichoic acids in the bacterial cell wall, influencing the physiology of the cells and their virulence [48]. Specifically, in Gram-positive bacteria, DltB is responsible for exporting D-alanine from the cell to the external environment, where it is incorporated into teichoic acids [49]. Modification of the teichoic acids with D-alanine can decrease the negative charge of the cell envelope. Consequently, this reduced negative charge can make it more difficult for cationic antimicrobial peptides (CAMPs) to interact with and disrupt the cell envelope [50]. In Gram-negative bacteria, it was shown that D-alanine may modify the lipid A components of the LPS to alter the global negative charge of their surface [51], leading to a similar effect as observed in Gram-positive bacteria. Overall, D-alanylation helps bacteria evade the host immune system. Alterations in the cell’s surface due to D-alanylation can also impact the ability of the bacterium to adhere to host tissues and form biofilms [52]. Interestingly, in *D. dadantii* strain 3937, genes involved in D-alanylation were reported to be up-regulated in cells in contact with aphids, suggesting their role in a strong response against antimicrobial peptides produced either by the aphids themselves or the aphid microbiota [53]. It may be that D-alanylation helps *D. solani* overcome *S. dulcamara* immune responses during the early stages of infection. However, more work is needed to understand the molecular link between D-alanylation and *D. solani* virulence in secondary plant hosts.

The gene encoding the YheO/PAS domain-containing putative transcription regulator was mutated in mutant M281. Knowledge about the specific function of the YheO/PAS domain-containing protein in SRP bacteria is limited. However, it can be assumed that it is similar to other transcriptional regulators, such as YheO that is accountable for controlling gene expression in response to environmental signals, including temperature, pH, nutrient availability, or the presence of toxins [54,55]. Some support for the hypothesis that transcriptional regulators can act as virulence factors has previously been reported. In *D. dadantii* strain 3937, several transcriptional regulators were reported to be involved in the infection process in different hosts [56].

Mutant M278 contained a disrupted gene encoding a glycosyltransferase. Glycosyltransferases allow pathogenic bacteria to modify their surface structures in a way to enhance their ability to infect, survive, and evade the host immune system [57,58]. These enzymes play a crucial role in bacterial virulence by modifying bacterial surface structures, such as lipopolysaccharides (LPS) and capsular polysaccharides. Glycosyltransferases are involved in LPS and EPS synthesis in plant pathogenic and plant-beneficial bacteria. For example, these enzymes were involved in synthesizing LPS and EPS in plant pathogenic *Xanthomonas citri* subsp. *citri* [59], as well as the synthesis of EPS in the plant-beneficial bacterium *Rhizobium* sp. YAS34, where the activity of this gene was critical for cell adherence to plant roots during host colonization [60].

In mutant M264, the gene encoding a hypothetical protein was disrupted. This gene is a member of an operon encoding three hypothetical proteins that is located between genes involved in basic bacterial metabolism. Neither the function of the mutated gene nor the function of the operon could be assessed.

Among the 8 genes identified in the study as linked to *D. solani* virulence on *S. dulcamara*, 6 were found within operons, while the remaining two were detected as individual transcripts. This suggests that the reduced virulence observed in these mutants may not solely result from the disruption of individual genes but also from the inactivation of other downstream genes. Though the influence of Tn5 transposition in *Dickeya* spp. has not been extensively studied to date [61], and there is limited knowledge regarding the effects of such transposon insertions in *D. solani*, it is reasonable to assume that introducing the Tn5 transposon into the IPO 2222 chromosome could have such a polar effect on the transcription of genes. Our understanding of the molecular interactions between *D. solani* and plant tissues remains limited. To fully assess the molecular basis of the infection of *S. dulcamara* by *D. solani*, more work is needed to validate the role of each candidate gene in the infection process *in planta*. To do so, future work linking genotype with phenotype should include the construction of in-frame deletion mutants in the candidate genes in strain IPO 2222 and complementation assays.

Of eight *D. solani* virulence-related genes, four exhibited a notable plant-dependent gene induction when analyzed with qRT-PCR. However, to our surprise, the expression of three other selected genes was not up-regulated by the presence of plant tissue under our experimental conditions, even though some evidence had been seen in their original identification using Gus reporter transposons. Similar phenomena were reported by others [62]. Some of the inconsistencies in apparent inducibility can be attributed to variations in the methodological approaches employed. Comparing the induction patterns observed in the GUS assay used for screening *D. solani* Tn5 mutants and the qRT-PCR conducted on genes identified in the wild-type background is influenced by the strong context-dependent patterns of gene expression that are being measured, the very different nature of reporter genes and the instantaneous estimates of mRNA made by qPCR in reporting gene expression. GUS reporter gene products are relatively long-lived and the enzymatic activity measured in such assays cannot differentiate current from earlier gene expression. In contrast, the qRT-PCR results represented the current level of short-lived mRNA. It is conceivable that numerous genes regulated by plants in *D. solani* are governed by what are referred to as short-lived transcripts [63] or are only transiently induced upon interaction with plants. As a result, it is anticipated that a greater number of genes will show induction, as assessed by the GUS reporter gene assay, wherein the end-point positive reaction is observed irrespective of the specific time within the experimental timeframe when gene induction occurs. In contrast, a qRT-PCR assay, which offers a snapshot of target gene expression at a particular time, may detect a comparatively lower number of induced genes. An alternative and complementary strategy to identify bacterial genes specifically up-regulated in one but not in another plant species during infection would be Tn-seq [64]. Tn-seq involves the integration of transposon mutagenesis with high-throughput sequencing technologies, facilitating a systematic interrogation of the bacterial genome for genes essential for a particular process. Tn-seq has already proven to be a useful approach to studying *D. dadantii* genes required for colonization and survival in chicory plants [65], *D. solani* genes involved in colonization of lesions and roots of potato plants [66], as well as *D. dadantii* and *D. dianthicola* genes important for the bacterial growth in potato tubers [67].

Based on our results, we propose a hypothesis suggesting that Soft Rot *Pectobacteriaceae* (SRP), including *D. solani*, employ at least two distinct strategies to govern infections in diverse plant hosts. Our assumption posits that, on a primary host such as potato, SRP engages in a virulence strategy that induces evident and severe symptoms, ultimately leading to the host’s death—an approach we termed the “maximization attack strategy”. Conversely, when infecting secondary hosts like weeds, these bacteria might deploy an alternative virulence program, enabling them to remain unnoticed within the plants and survive until a more suitable host becomes available—an approach we termed the “stealth mode strategy”. Further molecular studies on the ecology and interactions of SRP bacteria and *D. solani* with secondary plant hosts are required to further assess the SRP virulence program.

## 4. Materials and Methods

The methods of this study are in accordance with relevant institutional, national, and international guidelines and legislation. This study protocol also complies with the IUCN Policy Statement on Research Involving Species at Risk of Extinction and the Convention on the Trade in Endangered Species of Wild Fauna and Flora.

### 4.1. Bacterial Strains, Plants, and Growth Media

*Dickeya solani* wild-type strain IPO 2222 (WT) [23] was grown in tryptone soya broth (TSB, Oxoid, Basingstoke, UK) or M9 minimal medium (MP Biomedicals, Chiba, Japan) supplemented with glucose (Sigma-Aldrich, St. Louis, MO, USA) (M9 + 0.4% glucose) [68] in shaken cultures (200 rpm) at 28 °C for 24–48 h. Solid bacterial cultures were cultivated on tryptone soya agar plates (TSA, Oxoid) or M9 minimal medium agar plates with glucose (M9 + 0.4% glucose, supplemented with 15 g L^−1^ bacteriological agar (Oxoid)) at 28 °C for 24–48 h.

The *D. solani* Tn5 mutants (Supplementary Table S1) were previously described [22]. These mutants exhibited altered gene expression solely in contact with *S. dulcamara* plants but not in potato. The *D. solani* Tn5 mutants were cultured in the same media and under the same conditions as the wild-type strain, but the growth media were additionally supplemented with neomycin (Sigma-Aldrich) to the final concentration of 50 μg mL^−1^. In vitro plantlets of *S. dulcamara* (accession: A54750008, The Experimental Garden and Genebank, Radboud University, Nijmegen, The Netherlands) were acquired from Prof. Titti Mariani (Department of Molecular Plant Physiology, Radboud University, Nijmegen, The Netherlands). Plants were cultivated on Murashige and Skoog (MS) medium [69] supplemented with Gamborg’s vitamin mixture (Duchefa Biochemie bv., Haarlem, The Netherlands) (MS + G vit.), 30 g L^−1^ sucrose (Chempur, Pretoria, South Africa), pH 5.8, and solidified with 7 g L^−1^ plant agar (Duchefa Biochemie bv.) as described earlier [24]. The growth conditions involved maintaining them in an SMC-250-CC phytochamber (Sanwood Technologies, Dongguan, China) equipped with photosynthetic light banks (Sanwood D65, 15 W, 8500 K) under a 16/8 h light/dark regime, at a temperature of 22 ± 0.5 °C, as previously detailed elsewhere [24].

### 4.2. Determination of the Tn5 Insertion Sites through Genome Sequencing and Assessing the Functionality of the Disrupted Genes

The bacterial genomes were sequenced to identify the Tn5 insertion sites in the genomes of *D. solani* mutants induced in the presence of *S. dulcamara* tissues, as described earlier [22]. Structural and functional annotations in the obtained draft genome were acquired through RAST (Rapid Annotation using Subsystem Technology) (http://rast.nmpdr.org/ accessed multiple times in May 2022) [70]. The Tn5 insertion sites within the draft genomes of *D. solani* IPO2222 Tn5 mutants were determined through BlastN and BlastX alignments (http://blast.ncbi.nlm.nih.gov/Blast.cgi, accessed multiple times in May and June 2022) [71], using the complete genome sequence of *D. solani* strain IPO 2222 [72] as a reference. Detailed localization of the Tn5 transposon within the bacterial chromosome was assessed. For each mutant, sequences flanking the Tn5 insertion, ranging from approximately 1000 to 5000 base pairs, were analyzed to elucidate the genomic context of each Tn5-disrupted gene as described earlier [73]. The putative functions of the disrupted genes were inferred using BlastN and BlastX alignments. Additionally, the functions of target genes, including open reading frames (ORFs) encoding hypothetical proteins or proteins lacking homology to known proteins, were analyzed using the GeneSilico Protein Structure Prediction meta-server, incorporating known three-dimensional (3D) protein structures [74]. Likewise, PSI-BLAST [75], accessed from NCBI, was utilized in this analysis. Predicted functions with the highest scores were considered the most probable.

The presence of conserved domains in hypothetical proteins was evaluated using The Conserved Domain Database accessed via NCBI (https://www.ncbi.nlm.nih.gov/Structure/cdd/wrpsb.cgi, accessed in January 2024) [76]. The presence of protein homologs in different SRP and other bacterial species was evaluated using NCBI-BLASTP as described earlier [22].

### 4.3. Prediction of Transcriptional Organization and Analysis of Biochemical Pathways and Cellular Enzymatic Networks for D. solani Genes with Tn5 Insertions

The transcriptional organization (individual gene transcript vs. operon-based transcript) of selected *D. solani* genes with Tn5 insertions was predicted using the BioCyc Genome Database Collection, accessible at https://biocyc.org/ [77], accessed multiple times in March–June 2023. Analysis of potential biochemical pathways involving these genes was conducted through KEGG [78]. Additionally, the proteins were assessed for their anticipated roles in cellular networks using STRING (Search Tool for Retrieval of Interacting Genes/Proteins) v11 (https://string-db.org/, accessed multiple times in March–June 2023). Based on the proteome of *D. solani* strain IPO 2222, this analysis provided crucial insights into the protein–protein interactions [79].

### 4.4. Phenotypic Characterization of D. solani Tn5 Mutants

*D. solani* Tn5 mutants were analyzed using the BIOLOG Phenotypic Microarray System, utilizing GEN III and EcoPlate 96-well microplates (Biolog Inc., Hayward, CA, USA). Each GEN III plate comprises 94 phenotypic tests, encompassing 71 carbon source utilization assays and 23 chemical sensitivity assays. EcoPlates contain 31 distinct complex carbon sources (www.biolog.com). Bacterial cultures were cultivated on TSA for 24 h at 28 °C and then resuspended in inoculation fluid (IF-A) for GEN III or 10 mM phosphate buffer (pH 7.0) for EcoPlate, using a sterile cotton swab. The suspension’s turbidity was adjusted to approximately 90% T using a spectrophotometer [A = log(%T)]. One hundred microliters of the suspensions were injected into each well of the microplates using a multichannel pipette [22]. The inoculated plates were developed for 24 h at 28 °C. Subsequently, the wells were observed for a color change, indicating a positive reaction. Likewise, color development was recorded using an Epoch2 microplate spectrophotometer (BioTek, Winooski, VT, USA) with a λ = 570 nm wavelength filter. Plates inoculated with the wild-type *D. solani* IPO 2222 strain served as controls. Selected *D. solani* Tn5 mutants were also screened for various phenotypic features using plate assays. These included biofilm formation [80], cell doubling time in rich and minimal media [81], motility [82], resistance to NaCl [83], growth under different temperatures (5, 8, 15, 28, 37 and 42 °C) [84], production of secreted enzymes (cellulases, pectinases, proteases) [22] and resistance to H_2_O_2_ [85].

### 4.5. Host Colonization and Virulence of D. solani Tn5 Mutants on S. dulcamara Plants Grown in Culture Tubes

Selected Tn5 mutants were evaluated for their capacity to colonize and induce symptoms in tissue-cultured *S. dulcamara* plants following established procedures [22]. Apical nodes of *S. dulcamara* plantlets, each possessing a minimum of two leaf pairs, were harvested from approximately one-month-old plant culture stocks and placed in individual culture tubes with new plant growth medium. After 14 days, when the plants reached a height of roughly 4 to 6 cm, with roots measuring at least 1 cm in length and exhibiting a minimum of 4 to 6 leaf pairs, they were selected for inoculation [24]. In vitro plants (total n = 10 per mutant, 5 plants per mutant per experiment, and the experiment replicated one time with the same setup) were subjected to infection with *D. solani* Tn5 mutants by inoculating bacterial cultures onto the base of each plant just near the surface of the MS medium, following previously described methods [86]. Each mutant was tested on ten plants grown in individual tubes of De Wit (Duchefa Biochemie bv.). The experiment was repeated once with the same setup. Inoculated plants underwent visual inspection for symptom development at 14 days post-inoculation (dpi), in accordance with earlier protocols [24,86]. Briefly, five classes of symptoms were recognized, that is: (1) no symptoms—when plants express neither visible disease symptoms nor visible root colonization; (2) root colonization—acknowledged by a distinct turbidity of the MS medium around the roots, starting from the site of inoculation and spreading down the roots; (3) root embrowning—recognized as a change of root(s) color from white/yellowish to brown/black; (4) blackleg-like symptoms—blackening and soft rotting of the stem near the inoculation point; and (5) plant death—when the plant haulm decomposed expansively, collapsed and the plant parts were difficult to distinguish from one another due to heavy rotting [24]. Similarly, at 14 dpi, the quantification of bacteria within stems was conducted as previously outlined [22].

### 4.6. Expression of Selected D. solani Genes Quantified Using qRT-PCR

Expression of *D. solani* genes, identified in selected Tn5 mutants, demonstrating reduced or no virulence in *S. dulcamara* as well as the reference gene *lpxC* [87] and *yhb* [22] was analyzed using qRT-PCR. To achieve this, overnight cultures of *D. solani* in M9 medium with 0.4% glucose were incubated for approximately 16 h at 28 °C with shaking (200 rpm). Cultures with a bacterial density of around 10^9^ CFU mL^−1^ were then diluted at a 1:50 (*v*/*v*) ratio in the same fresh medium. Subsequently, 20 milliliters of the diluted bacterial culture were aseptically moved to a sterile 50-mL Falcon tube (Sarstedt, Nümbrecht, Germany), and five 14-day-old *S. dulcamara* plants, each grown in individual culture tubes with roots, were added to the tube. The inoculated tubes were then incubated for 16 h at 28 °C with gentle shaking (50 rpm). Five independent cultures (=5 biological replicates) were processed per both the control (n = 5) and the treatment (n = 5). At the point of harvest, the IPO 2222 cultures grown in the presence of *S. dulcamara* plants were centrifuged at 1000 RCF for 1 min to clear the samples from plant debris. For all processed samples, 500 μL of bacterial suspension was mixed with 1 mL of Protect Bacteria reagent (QIAGEN, Venlo, The Netherlands), followed by incubation for 5 min. at room temperature (ca. 22 °C) and cell harvest by centrifugation (10 min., 5000 RCF). RNA was isolated from bacterial pellets using the RNeasy Mini Kit (QIAGEN). Potential contamination with gDNA was eliminated using the TURBO DNA-free™ Kit (Thermo Fisher Scientific, Waltham, MA, USA). The preserved bacterial pellets and the isolated RNA were stored at −80 °C between the subsequent processing steps. RNA was aliquoted to prevent multiple freeze–thaw cycles. Bacterial total RNA was reverse-transcribed to cDNA using Transcriptor First Strand cDNA Synthesis Kit (Roche, Basel, Switzerland) with random hexamer primers and the optional denaturation step. The amount of RNA per reaction was adjusted to 500 ng. The obtained cDNA was diluted 1:7 in sterile water. Real-time qPCR was performed in 96-well plates using the sample maximization design [88]. Each reaction mixture comprised 2× Power SYBR Green Mastermix (Thermo Fisher Scientific, USA), forward and reverse primers (300 nM each), and 4 μL of diluted cDNA as a matrix. The cycling protocol, implemented in a CFX96 instrument (Bio-Rad, Hercules, CA, USA), was as follows: 95 °C for 10 min, 40 cycles of 95 °C for 15 s, and 60 °C for 1 min, with a final melt curve of 55–95 °C (0.5 °C/5 s increment) [89]. Primers were designed using Primer3Plus [90]. The size of the amplicons was confirmed by gel electrophoresis. Primer efficiencies were established based on standard curves (5–7 points, with the highest R^2^). The *lpxC* and *yhb* were used as reference genes for data normalization [22,87]. For primer details, refer to Supplementary Table S3. Gene expression analysis, including statistics to verify the significance of differences between biological groups, was performed with the CFX Maestro 2.2 software, version 5.2.008.0222 (Bio-Rad). Normality of data was tested using the Shapiro–Wilk test [91]. Analysis of variance (ANOVA) was followed by the Tukey post hoc test (α = 0.05) [92].

### 4.7. Statistical Analyses

Bacterial counts underwent analysis through ordinary linear regression using the statistical software package Past version 4.13 [93]. To achieve normality, the data were transformed using log10 (x + 1). Significance was determined at *p* ≤ 0.05, and pair-wise differences were assessed using the *t*-test [94]. In experiments involving in vitro *S. dulcamara* plants, the data were examined based on an experimental design comprising two replicated experiments for each treatment of replicated plants. The chosen linear model followed a complete block design with replicates as blocks [95]. The main effects were analyzed to assess the contributions of time and treatment, along with the two-way interaction between time and treatment. A normal distribution of plant height and weight was assumed.

## 5. Conclusions

This study focused on understanding how necrotrophic SRP bacteria, particularly *D. solani*, adapt to and infect secondary plant hosts, specifically weed plants. Our study identified candidate genes present in the genome of *D. solani* and linked to *in planta* fitness and virulence during *S. dulcamara* infection. Likewise, our study, for the first time, highlighted the regulation of gene expression in response to *S. dulcamara*, with networks involved in antibiotic synthesis, polysaccharide formation, and membrane transport being crucial for the establishment of the disease and its progression in this host. Based on the obtained results, we propose the existence of distinct virulence strategies employed by *D. solani* (and probably by other members of Soft Rot *Pectobacteriaceae*), namely the “maximization attack strategy” on primary hosts and the “stealth mode strategy” on secondary hosts, urging further molecular investigations into SRP virulence programs on different plants and under various conditions.

## Data Availability

The datasets used and analyzed during the current study are available from the corresponding author upon reasonable request.

## Supplementary Materials

The following supporting information can be downloaded at: https://www.mdpi.com/article/10.3390/ijms25052794/s1.
