# Supplementary material for: Genetic Loci of Plant Pathogenic Dickeya solani IPO 2222 Expressed in Contact with Weed-Host Bittersweet Nightshade (Solanum dulcamara L.) Plants
Source: Int J Mol Sci. 2024 Feb 28;25(5):2794. doi: 10.3390/ijms25052794 (PMC10931765; doi:10.3390/ijms25052794)
Supplement: Supplementary file 1 [file ijms-25-02794-s001.zip › ijms-2878291-Supplementary Tables.pdf]

Supplementary Tables

Supplementary Table S1. Genetic loci of *D. solani* strain IPO 2222 Tn5 mutants regulated in the presence of *S. dulcamara* tissues

No.	Mutant	Insertion name, Tn5 locus	Protein accession number, protein name	Transcriptional organization (single gene vs. operon)	Entry, KEGG pathway, UniProt-based protein function	Remarks	Homologs in different bacterial species
1	M69	***ds69***, A4U42_RS11770	WP_022632034, glycosyl transferase	operon, 12 genes: A4U42_RS11790, A4U42_RS11785, A4U42_RS11780, A4U42_RS11775, **A4U42_RS11770**, A4U42_RS11765, A4U42_RS11760, A4U42_RS11755, A4U42_RS11750, A4U42_RS11745, A4U42_RS11740, A4U42_RS11735	glycosyltransferase family 4 protein, capsular glucan synthase	in operon together with *ds271* and *ds943*	
2	M73	***ds73***, A4U42_ RS15630	WP_022632758, mannosyl transferase	single gene	glycosyltransferase family 4 protein		
3	M93	***ds93***, A4U42_ RS01565	WP_022634127, mycocerosate synthase	operon, 2 genes: **A4U42_RS01565**, A4U42_RS01560	SDR family NAD(P)-dependent oxidoreductase		
4	M106	***ds106***, A4U42_RS13890	WP_022632437 transcriptional regulator GcvA	single gene	regulation of transcription, LysR family transcriptional regulator, glycine cleavage system transcriptional activator		
5	M115	***ds115***, A4U42_RS16110	WP_022632856, non-ribosomal peptide synthase	operon, 8 genes: A4U42_RS16130, A4U42_RS16125, A4U42_RS16120, A4U42_RS16115, **A4U42_RS16110**, A4U42_RS16105, A4U42_RS16100, A4U42_RS16095	no KEGG pathway, non-ribosomal peptide synthase	in operon together with *ds255*	

6	M119	***ds119***,<br>A4U42_RS01450	WP_022634107<br>FMN/FAD transporter	single gene	EmmdR/YeeO family multidrug/toxin efflux MATE transporter		
7	M241	***ds241***, ***tgt***<br>A4U42_RS14365	WP_022632529,<br>tRNA guanosine transglycosylase Tgt	operon, 2 genes:<br>*queA*,<br>*tgt*	queuine tRNA-ribosyltransferase, tRNA guanosine transglycosylase Tgt		
8	M253	***ds253***, ***wbeA***<br>A4U42_RS06140	WP_022634991<br>WbeA	operon, 7 genes:<br>*gmd*<br>**A4U42_RS06140**<br>A4U42_RS06135<br>A4U42_RS06130<br>A4U42_RS06125<br>A4U42_RS06115<br>A4U42_RS06110	entry not assigned,		
9	M255	***ds255,***<br>A4U42_RS16105	WP_022632855,<br>non-ribosomal peptide synthetase	operon, 8 genes:<br>A4U42_RS16130,<br>A4U42_RS16125,<br>A4U42_RS16120,<br>A4U42_RS16115,<br>A4U42_RS16110,<br>**A4U42_RS16105**,<br>A4U42_RS16100,<br>A4U42_RS16095	non-ribosomal peptide synthetase	in operon together with *ds115*	
10	M264	***ds264,***<br>A4U42_RS11855	WP_022632050,<br>hypothetical protein	operon, 3 genes:<br>**A4U42_RS11855**,<br>A4U42_RS11850,<br>A4U42_RS11845	entry not assigned, contains endonuclease subunit and chromosome segregation ATPase subunit		
11	M271	***ds271,***<br>A4U42_RS11740	WP_023637586,<br>capsular biosynthesis protein	operon, 12 genes<br>A4U42_RS11790,<br>A4U42_RS11785,<br>A4U42_RS11780,<br>A4U42_RS11775,<br>A4U42_RS11770,<br>A4U42_RS11765,<br>A4U42_RS11760,<br>A4U42_RS11755,<br>A4U42_RS11750,<br>A4U42_RS11745,	polysaccharide biosynthesis protein VpsM, outer membrane beta-barrel protein	in operon together with *ds69* and *ds943*	

				**A4U42_RS11740**,<br>A4U42_RS11735			
12	M273	***ds273, bglF***<br>A4U42_RS00580	WP_022633945<br>PTS beta-glucoside transporter subunit IIABC	operon, 3 genes:<br>*bglG*,<br>***bglF***,<br>*ascB*	beta-glucoside PTS system EIICBA component [EC:2.7.1.-]		
13	M277	***ds277,***<br>A4U42_RS04070	WP_022634595,<br>DltB protein for D-alanine esterification of lipoteichoic acid and wall teichoic acid	operon, 3 genes:<br>**A4U42_RS04070**,<br>A4U42_RS04065,<br>A4U42_RS04060	membrane protein involved in D-alanine export,<br>DltB protein for D-alanine esterification of lipoteichoic acid and wall teichoic acid		
14	M278	***ds278,***<br>A4U42_RS02215	WP_022634251<br>glycosyltransferase	single gene	glycosyltransferase, cellulose synthase catalytic subunit (UDP-forming)		
15	M281	***ds281,***<br>A4U42_RS17100	WP_022633056,<br>predicted transcriptional regulator YheO	single gene	PAS domain-containing protein		
16	M321	***ds321,***<br>A4U42_RS16640	WP_022632961,<br>D-cysteine desulfhydrase	operon, 2 genes:<br>**A4U42_RS16640**<br>A4U42_RS16645	L-cysteate sulfo-lyase [EC:4.4.1.25]		
17	M327	***ds327,***<br>A4U42_RS19655	WP_022633559,<br>ClbS/DfsB family four-helix bundle protein	single gene	ClbS/DfsB family four-helix bundle protein		
18	M328 and M880	***ds328,***<br>A4U42_RS17270	WP_022633089,<br>glucarate transporter	single gene	MFS transporter, ACS family, D-galactonate transporter		
19	M468	***ds468,***<br>A4U42_RS17435	WP_022633124,<br>conjugal transfer protein TraR	operon, 3 genes:<br>A4U42_RS17445,<br>A4U42_RS17440,<br>**A4U42_RS17435**	TraR/DksA family transcriptional regulator		
20	M519	***ds519, nifN***<br>A4U42_RS06315	WP_022635026,<br>nitrogen fixation protein NifN	operon, 3 genes :<br>*nifE*<br>***nifN***<br>A4U42_RS06310	nitrogenase molybdenum-iron protein NifN		
21	M521	***ds521,***<br>A4U42_RS16075	WP_223849466,<br>polyketide synthase	single gene	SDR family NAD(P)-dependent oxidoreductase		
22	M564	***ds564,***<br>A4U42_RS04280	WP_022634635,<br>nitrate reductase	single gene	nucleobase:cation symporter-1, NCS1 family		

23	M573	***ds573,***<br>A4U42_RS11730	WP_022632027,<br>undecaprenyl-phosphate glucose phosphotransferase	single gene	L-talarate/galactarate dehydratase		
24	M578	***ds578,***<br>A4U42_RS17980	WP_023637770,<br>peptide permease	operon, 4 genes:<br>A4U42_RS17995,<br>A4U42_RS17990,<br>A4U42_RS17985,<br>**A4U42_RS17980**	peptide/nickel transport system permease protein, ABC transporter permease		
25	M585	***ds585,***<br>A4U42_RS07980	WP_022635320,<br>phage tail protein	operon, 2 genes:<br>**A4U42_RS07980**<br>A4U42_RS07975	phage tail protein		
26	M596	***ds596, uvrY (expA, gacA)***<br>A4U42_RS01590	WP_022634131,<br>two-component system response regulator UvrY (GacA)	operon, 2 genes:<br>*uvrY,*<br>*uvrC*	two-component system, NarL family, invasion response regulator UvrY, two component system ExpA-ExpS in SRP bacteria		
27	M598 and M980	***ds598,***<br>A4U42_RS08155	WP_022635352,<br>malate permease	single gene	malate:Na+ symporter, 2-hydroxycarboxylate transporter family protein		
28	M620 and M940	***ds620, dprA***<br>A4U42_RS06665	WP_022635094,<br>DNA-protecting protein DprA	single gene	DNA processing protein, DNA-protecting protein DprA		
29	M622	***ds622, prfC***<br>A4U42_RS11580	WP_022631998,<br>peptide chain release factor 3	single gene	peptide chain release factor 3		
30	M635	***ds635, menH***<br>A4U42_RS14815	WP_023637673,<br>2-succinyl-6-hydroxy-2,4-cyclohexadiene-1-carboxylate synthase	operon, 6 genes:<br>*menF*<br>*menD*<br>***menH***<br>*menB*<br>*menC*<br>*menE*	2-succinyl-6-hydroxy-2,4-cyclohexadiene-1-carboxylate synthase [EC:4.2.99.20]		
31	M652	***ds652, sctR***<br>A4U42_RS20675	WP_022633761,<br>EscR/YscR/HrcR family type III secretion system export apparatus protein	operon, 11 genes:<br>*sctW*<br>*sctV*<br>*sctD*<br>*scN*<br>***sctR***<br>*sctS*<br>*sctT*	type III secretion protein R,<br>type III secretion system export apparatus subunit SctR		

				sctU<br>A4U42_RS20670<br>A4U42_RS20665<br>A4U42_RS20660			
32	M702	***ds702,***<br>A4U42_RS13765	WP_230469717,<br>serine protease	single gene	subtilase-type serine protease [EC:3.4.21.-], autotransporter domain-containing protein		
33	M828	***ds828,***<br>A4U42_RS16290	WP_022632893,<br>nickel ABC transporter permease subunit NikC	operon, 4 genes:<br>A4U42_RS16300,<br>A4U42_RS16295,<br>**A4U42_RS16290**,<br>A4U42_RS16285	nickel transport system permease protein, ABC transporter permease subunit		
34	M854	***ds854,***<br>A4U42_RS09805	WP_022631675,<br>glutathione ABC transporter permease GsiC	operon, 5 genes:<br>A4U42_RS09810,<br>**A4U42_RS09805**,<br>A4U42_RS09800,<br>A4U42_RS09795,<br>A4U42_RS09790	glutathione transport system permease protein		
35	M884	***ds884,***<br>A4U42_RS00600	WP_022633948,<br>peptidoglycan-binding protein	single gene	L,D-transpeptidase YcfS		
36	M890	***ds890,***<br>A4U42_RS09520	WP_022631619,<br>pseudouridine-5-phosphate glycosidase	operon, 2 genes:<br>**A4U42_RS09520**<br>A4U42_RS09525	pseudouridylate synthase [EC:4.2.1.70],<br>pseudouridine-5'-phosphate glycosidase		
37	M937	***ds937, glpX***<br>A4U42_RS07915	WP_022635307,<br>fructose 1,6-bisphosphatase	operon, 3 genes:<br>*glpK*<br>***glpX***<br>A4U42_RS07905	fructose-1,6-bisphosphatase II [EC:3.1.3.11],<br>class II fructose-bisphosphatase		
38	M943	***ds943,***<br>A4U42_RS11735	WP_023637585,<br>undecaprenyl-phosphate glucose phosphotransferase	operon, 12 genes:<br>A4U42_RS11790,<br>A4U42_RS11785,<br>A4U42_RS11780,<br>A4U42_RS11775,<br>A4U42_RS11770,<br>A4U42_RS11765,<br>A4U42_RS11760,<br>A4U42_RS11755,<br>A4U42_RS11750,	undecaprenyl-phosphate glucose phosphotransferase	in operon together with *ds271* and *ds69*	

				A4U42_RS11745,<br>A4U42_RS11740,<br>**A4U42_RS11735**			
39	M945	***ds945,***<br>A4U42_RS12920	WP_022632248,<br>ferredoxin	operon, 4 genes:<br>A4U42_RS12930,<br>A4U42_RS12925,<br>**A4U42_RS12920**,<br>A4U42_RS12915	ferrodoxin,<br>2Fe-2S iron-sulfur cluster-binding protein		
40	M946	***ds946,***<br>A4U42_RS12865	WP_022632237,<br>aminotransferase	operon, 2 genes:<br>**A4U42_RS12865**<br>A4U42_RS12870	putrescine aminotransferase [EC:2.6.1.82],<br>aminotransferase class III-fold pyridoxal phosphate-dependent enzyme		
41	M955	***ds955, fdhF***<br>A4U42_RS16455	WP_022632925,<br>formate dehydrogenase subunit alpha	operon, 2 genes:<br>***fdhF***<br>A4U42_RS16460	formate dehydrogenase (hydrogenase) [EC:1.17.98.4 1.17.98.-],<br>formate dehydrogenase subunit alpha		
42	M1009	***ds1009,***<br>A4U42_RS16195	WP_022632873,<br>ABC transporter	single gene	ATP-binding cassette, subfamily F, member 3		

Supplementary Table S2. Results of BIOLOG phenotypic microarrays GEN III and EcoPLate for 8 mutants expressing no or reduced symptoms on *S. dulcamara* planta

**GEN III**	**IPO2222**	**M241**	**M253**	**M264**	**M271**	**M277**	**M278**	**M281**	**M596**
D-cellobiose	+	+	-	-	+	+	+	+	+
D-turanose	-	-	-	+	-	-	-	-	-
L-glutamic acid	+	-	-	+	-	+	+	+	+
D-glucuronic acid	+	+	-	+	-	+	+	+	+
pH 5	+	+	+	+	+	+	-	+	+
4% NaCl	-	-	-	-	-	+	-	-	+
8% NaCl	-	-	-	-	-	-	-	-	+
fusidic acid	-	+	-	-	-	+	-	-	+
D-serine	+	+	-	-	-	-	-	-	-
guanidine HCl	+	+	-	+	+	+	+	+	-
lithium chloride	+	+	+	+	-	+	-	+	+
sodium butyrate	-	+	-	-	-	+	-	-	+
**EcoPlate**	**IPO2222**	**M241**	**M253**	**M264**	**M271**	**M277**	**M278**	**M281**	**M596**
D-xylose	+	+	+	+	+	+	+	-	+
L-asparagine	+	+	+	+	+	+	+	-	+
D-mannitol	+	+	+	+	+	+	-	+	+
L-serine	+	+	+	+	-	+	+	+	+
D-malic acid	-	-	-	+	+	+	+	-	+

“+” – positive result (=change of medium color due to bacterial growth and metabolism)

“-” – negative result (=the lack of change of medium color = no bacterial growth/no bacterial metabolism)

Supplementary Table S3. List of PCR primers used in this study with amplification efficiencies in the applied setup

Target locus	Primer name [A]	Primer sequence	Amplicon length (bp)	E[B]	R^2	Slope	Reference
A4U42_14370	7_M241_F	TATTTGATGGGGGTGGGTAA	150	102.1%	0.998	-3.272	This study
	7_M241_R	GTGTTTGGCGTTACGGATCT					
A4U42_06145	8_M253_F	AGCCGGACTCTATGGCACTA	146	97.5%	0.990	-3.384	This study
	8_M253_R	GAGCCTGTTTAATGGCTTCG					
A4U42_11860	10_V2_M264_F	AAGTGTGTTTGTGGGCATGA	121	100.5%	0.992	-3.311	This study
	10_V2_M264_R	GAGCGACTTCCATCGTCTCT					
A4U42_11745	11_M271_F	TAGGCCGCACAGAGGATATT	140	90.1%	0.997	-3.585	This study
	11_M271_R	CATAAAGCGGTAGCGAAAGC					
A4U42_04075	13_M277_F	GCGCAGAATATTGGCATTTT	127	74.3%	0.997	-4.146	This study
	13_M277_R	ACCACTGAAGCATGTTGTGC					
A4U42_02220	14_M278_F	CTGGGATCACGTTTTCGTTT	132	74.9%	0.996	-4.118	This study
	14_M278_R	TGCAATATAATCGGCATCCA					
A4U42_17105	15_M281_F	GTCGCGTCTCTGTGTCTCAA	147	90.6%	0.973	-3.568	This study
	15_M281_R	CTGATCGATACGCTGACGAA					
A4U42_01595	26_M596_F	CGGTGAAGATGCGGTAAGAT	108	93.8%	0.990	-3.479	This study

	26_M596_R	CGTACAATTTTGCGTGTTGC					
A4U42_03695	V2_yhb_F	CAGCACATGGGTCAACAAAC	126	91.1%	0.997	-3.555	This study
	V2_yhb_R	ACATGTACGATTCCGCATCA					
A4U42_05475	lpxC_F	ATGAACAGGTCGCCAATCG	93	84.2%	0.999	-3.771	PMID: 32983224
	lpxC_R	AGATGATTACCGTGTGCTGAAC					

[A] designation of the source Tn mutant is included in the primer name for all genes except for the reference genes *lpxC* and *yhbN*

[B] primer pair efficiency
